# Comparison of Magnesium Pre-treatment With Two Different Doses of Rocuronium in Rapid Sequence Intubation: A Randomized Controlled Trial

**DOI:** 10.7759/cureus.56794

**Published:** 2024-03-23

**Authors:** Mudita Sharma, Ravi Prakash, Manoj K Chaurasia, Rati Prabha, Rajesh Raman, Gyan P Singh, Gauri Arora

**Affiliations:** 1 Anesthesiology and Critical Care, King George's Medical University, Lucknow, IND

**Keywords:** muscle relaxant, rapid sequence intubation (rsi), onset time, rocuronium, magnesium pre-treatment

## Abstract

Introduction

Magnesium is recognized for its ability to reduce the onset time of rocuronium while simultaneously extending its duration of action. This study aims to assess the efficacy of magnesium pre-treatment in decreasing the onset time with two different doses of rocuronium in patients undergoing rapid sequence intubation.

Materials and methods

This randomized prospective double-blind clinical study involved 50 patients classified as American Society Of Anesthesiologists (ASA) I/II, with no preoperative indications of difficult intubation, undergoing elective surgery under general anesthesia. The patients were divided into two groups: group A received 60 mg/kg of magnesium 15 minutes before intubation with 1.2 mg/kg of rocuronium, and group B received 60 mg/kg of magnesium before 0.6 mg/kg of rocuronium. Intubating conditions were assessed and graded at loss of last twitch after administration in both groups, considering ease of intubation, vocal cord position, and response to the insertion of the tracheal tube. Simultaneously, hemodynamic variations were recorded just before intubation, at one minute and five minutes post-intubation.

Results

Intubating conditions with 0.6 mg/kg of rocuronium were comparable or equally good compared to 1.2 mg/kg of rocuronium with magnesium pre-treatment.

Conclusions

Magnesium pre-treatment enhances the neuromuscular blocking effect of rocuronium, reducing its onset time without clinically significant prolongation of the duration of the block.

## Introduction

Rapid sequence intubation (RSI) is necessary for patients at risk of aspiration due to various reasons [1]. It conventionally involves preoxygenation, induction with a fixed dose of an induction agent, followed by a rapidly acting muscle relaxant, and intubation within one minute without positive pressure ventilation. Sympathetic stimulation can lead to increased blood pressure and heart rate, while inadequate neuromuscular blockade results in coughing and spasms of vocal cords [2].

Succinylcholine was the sole agent used for RSI due to its rapid onset and short action. However, it comes with multiple side effects, such as muscle soreness, hyperkalemia, bradycardia, and elevated pressures in the eye, stomach, and brain. Additionally, it can lead to malignant hyperthermia in susceptible individuals and may cause prolonged apnea in patients with pseudocholinesterase deficiency [3,4].

Rocuronium and mivacurium are two drugs that produce effects comparable to succinylcholine. Rocuronium, an amino-steroid non-depolarizing neuromuscular blocking agent, yields clinically acceptable intubating conditions within 60 to 90 seconds at a dose of 0.6 mg/kg, similar to succinylcholine, with a duration of 30-40 minutes [5,6].

Magnesium sulfate (MS) has found use in anesthesia due to its unique qualities such as analgesia, membrane stabilization, and muscle relaxation [7]. MS potentiates neuromuscular blockade and has anti-adrenergic action, thus suppressing the response to laryngoscopy and intubation [8]. The onset of rocuronium is shortened by pre-treatment with MS, and patients treated with magnesium exhibit stable hemodynamics with lower plasma concentrations of catecholamines during tracheal intubation [9-11].

The primary objective of the present study was to measure the efficacy of magnesium pre-treatment in reducing the onset time with two different doses of rocuronium in patients undergoing RSI. Secondary objectives were to assess the difference in intubating conditions and the duration of action of the intubating dose.

## Materials and methods

This is a randomized prospective double-blind comparative study. It was conducted in a tertiary care center for one year. The sample size formulae used are as follows [12]: n = sample size, σ = standard deviation, ∆ = difference of means, κ = ratio, Z1-α/2 = two-sided Z value, Z1-β = power, confidence interval = 95%, power = 90%. Taking the study by Mohammed et al. [13] as a reference, our minimum sample size is calculated as 42; hence, we incorporated 50 patients in our study (25 in each group).

After obtaining ethical approval from the institutional ethical committee (1969/ethics/2022), the clinical trial was registered at the Clinical Trials Registry of India (CTRI; Trial REF/2022/01/051187). Written and informed consent was obtained from all participants. Patients of either gender, aged 18-65 years, with an American Society of Anesthesiologists (ASA) physical status I or II, scheduled for elective surgery lasting longer than 60 minutes under general anesthesia, Mallampati grade I/II, with mouth opening of at least 3 cm, and a body mass index in the normal range (18.5-24.9), were enrolled. They were divided into two groups using a computer-generated random number table. Group A received 60 mg/kg of magnesium pre-treatment in 100 ml saline followed by 1.2 mg/kg of rocuronium, while group B received 60 mg/kg of magnesium pre-treatment in 100 ml saline followed by 0.6 mg/kg of rocuronium.

Blinding was ensured by allowing the intubating anaesthesiologist to enter the operating theatre only after drug administration. After securing intravenous access and attaching standard monitors, patients were preoxygenated, and magnesium was administered (100 ml infusion) over 15 minutes. Neuromuscular monitoring was conducted with a train-of-four (TOF) watch (Drager, Lübeck, Germany). Induction was performed with 2 mcg/kg of fentanyl and 1-2 ml/kg of propofol. TOF monitoring commenced. Rocuronium was administered as a bolus according to the allocated group. The time interval between the administration of the intubating dose of rocuronium and the loss of the last twitch on TOF was considered the onset time. The time of recovery of the first twitch of TOF from the onset was considered as the duration of action or the requirement for the first maintenance dose. Intubation was attempted after the loss of the last twitch on TOF monitoring, and intubating conditions were graded as excellent, good, or poor using a modified scale based on the recommendations of good clinical research practice in neuromuscular research (Table 1). Heart rate and blood pressure were recorded before intubation, at one minute, and five minutes after intubation. The data was entered in Microsoft Excel (Microsoft, Redmond, Washington) and analyzed using statistical software SPSS version 25 (IBM Inc., Armonk, New York)

**Table 1:** Intubating conditions according to good clinical practice in neuromuscular research

Variable assessed	Clinically acceptable		Not clinically acceptable
	Excellent	Good	Poor
Laryngoscopy	Easy (jaw relaxed, no resistance to blade insertion)	Fair (jaw not fully relaxed, slight resistance to blade insertion)	Difficult (poor jaw relaxation, active resistance of the patient to laryngoscopy)
Vocal cords position	Abducted	Intermediate/moving	Closed
Reaction to insertion of the tracheal tube and cuff inflation (Diaphragmatic movement/coughing)	None	Slight (One to two weak contractions and/or movement for <5 s)	Vigorous/sustained (More than two contractions and/or movement for longer than 5 s)

Student's t-test was used to test the significance of the difference between quantitative variables and Fisher's Chi-squared test for qualitative variables. A p-value less than 0.05 was taken to denote a significant relationship.

## Results

A total of 55 patients were screened, out of which 50 patients were enrolled in the study. They were randomly divided into two groups (n=25). No patients were excluded from the study after enrollment. The difference between the study population was found to be statistically insignificant regarding gender distribution and mean age comparison (Table 2).

**Table 2:** Distribution of the studied patients on the basis of their gender and age Student's t-test p<0.05 is significant

Demographic variables	Group A (n=25)	Group B (n=25)	p-value
Male	10 (40.0)	12 (48.0)	0.568
Female	15 (60.0)	13 (52.0)	
Mean age (years)	37.12±12.3	33.6±12.9	0.328

The onset time of rocuronium, i.e., time between injection of rocuronium and loss of last twitch on TOF watch, was significantly lower in group A as compared to group B. The duration of action of rocuronium is significantly higher in group A as compared to group B (Table 3).

**Table 3:** Onset time and duration of action or requirement of first maintenance dose of rocuronium Student's t-test p<0.05 is significant

Onset time (sec)	Group A (n=25)	Group B (n=25)	p-value
Range	44-58 sec	52-68 sec	<0.001
Mean±SD	51.95±5.81	60.57±5.82	
Duration of action/ requirement of 1st maintenance dose (min)	40.04±3.61	25.92±3.9	<0.001

The following table shows the distribution of the studied patients based on laryngoscopy, wherein it was found that it was easy in 96.0% of patients of group 2 and 72.0% of patients of group 1, and none of the patients faced difficulty in both of the groups. Vocal cord position was abducted in 80.0% in group 2 and 72.0% in group 1, and reaction to insertion of tracheal tube/cuff inflation was none in both groups (Table 4).

**Table 4:** Distribution of the studied patients based on laryngoscopy and vocal cord position p<0.05 is significant Fisher's exact test

Laryngoscopy and vocal cord insertion		Group A (n=25)	Group B (n=25)	p-value
Laryngoscopy	Easy	18 (72.0%)	24 (96.0%)	0.068
	Fair	7 (28.0%)	1 (4.0%)	
	Difficult	0 (0.0)	0 (0.0)	
Vocal cord position	Abducted	18 (72.0%)	20 (80.0%)	0.802
	Intermediate	7 (28.0%)	5 (20.0%)	
	Closed	0 (0.0)	0 (0.0)	
Reaction to insertion of tracheal tube/cuff inflation	None	25 (100.0%)	25 (100.0%)	1.00
	Slight	0 (0.0)	0 (0.0)	
	Vigorous	0 (0.0)	0 (0.0)	

Comparison of systolic-diastolic blood pressure and heart rate did not reveal any significant differences in both groups (Table 5).

**Table 5:** Comparison of systolic and diastolic blood pressure in both groups SBP - systolic blood pressure; DBP - diastolic blood pressure, Student's t-test

Time of observation	Group A (n=25)			Group B (n=25)			p-value
	SBP (mm Hg)	DBP (mm Hg)	H.R.	SBP (mm Hg)	DBP (mm Hg)	H.R.	
Before intubation	124.08±13.7	79.28±9.9	76.8±10.6	117.96±13.7	77.76±8.4	77.8±12.5	0.475
One minute post-intubation	112.40±2.6	83.36±8.03	84.2±11.5	113.7±3.10	80.64±8.6	85.4±11.9	0.359
Five minutes post-intubation	123.20±14.3	80.56±7.8	82.4±11.2	117.20±13.1	76.96±8.1	81.4±11.9	0.989

## Discussion

The priming technique consists of administrating a small dose of neuromuscular blocking agent minutes prior to induction, allowing sufficient time for the relaxant to reach the receptors, and then administering a second larger dose to facilitate rapid intubation after induction. The priming principle can be applied to rocuronium with an intubating dose of 0.6 mg/kg. A priming dose of 0.06 mg/kg (10.0% of intubating dose) and a priming interval of three minutes can be used in order to achieve an onset of action closer to succinylcholine (54 seconds) [8,15,16]. Higher doses of rocuronium are required for effective action and may provide a very similar onset time to that of succinylcholine. Nonetheless, several strategies have been implemented to reduce the onset time of rocuronium, including the use of magnesium sulfate (MgSO4) [16]. However, the use of high doses of rocuronium, especially when associated with magnesium sulfate, may significantly prolong the duration of neuromuscular blockade [16]. The onset of action, from the administration of rocuronium until 95.0% suppression of the first twitch, is faster at higher doses of rocuronium and is shortened by pre-treatment with MgSO4. Second, MgSO4 has anti-adrenergic effects by decreasing catecholamine release from the adrenal medulla or adrenergic nerve endings, and it causes vasodilation and an anti-arrhythmic effect on the heart [14]. Magnesium-treated patients show more stable arterial pressure and heart rate with a lower plasma concentration of catecholamines during tracheal intubation.

The introduction of rocuronium represents a groundbreaking development, offering onset times and intubating conditions similar to succinylcholine but without its associated side effects. The availability of the reversal agent sugammadex further expands its utility, even in burn patients and those with challenging intubation conditions. Despite rocuronium's delayed onset compared to succinylcholine, this can be mitigated by increasing the dose to 1.2 mg/kg in rapid sequence intubation (RSI). However, this higher dose results in a prolonged duration of action, rendering it unsuitable for shorter surgeries. The lack of sugammadex availability makes it unsafe in difficult airway conditions with such an extended duration of action. Magnesium pre-treatment facilitates early endotracheal intubation, even with a lower dose of rocuronium (0.6 mg/kg), similar to RSI with the 1.2 mg/kg dose and without side effects. Patients receiving magnesium pre-treatment exhibited excellent intubation conditions, such as ease of laryngoscopy and vocal cord positioning, even with the lower dose, compared to the higher dose of 1.2 mg/kg. The hemodynamic variations in blood pressure and heart rate during laryngoscopy and intubation were attenuated with clinically insignificant side effects, making the magnesium-rocuronium combination valuable in trauma and burn patients where major hemodynamic changes are undesirable. Although magnesium pre-treatment extends the duration of action of rocuronium, it does so to a clinically insignificant extent, avoiding complications arising from prolonged neuromuscular blockade [11].

In our study, the mean onset time in group A, which received 1.2 mg/kg of rocuronium, was 51.9 seconds, while group B, which received 0.6 mg/kg of rocuronium, had a mean onset time of 60.5 seconds. Magnesium pre-treatment reduced the onset time of rocuronium such that even a lower dose of 0.6 mg/kg had an onset time almost equal to that of a much higher dose of 1.2 mg/kg. Although the difference in onset time is statistically significant, it is comparable to succinylcholine with no clinically relevant difference. A study by Sun et al. [11], analyzing eleven RCTs, concluded that adding magnesium sulfate before rocuronium during general anesthesia shortened its onset time and prolonged its clinical duration without significantly increasing the recovery time.

Our study assessed intubating conditions with three variables, including laryngoscopy, vocal cord position, and reaction to tube insertion, and found them to be excellent/good in both groups. A similar study by Park et al., involving 154 patients divided into three groups (rocuronium 0.6 mg/kg, rocuronium 0.9 mg/kg, and the third group with magnesium pre-treatment followed by either dose), concluded that intubating conditions improved significantly in the magnesium group and also prevented post-intubation hypertension [2].

Our study further concluded that magnesium pre-treatment, even with a lower dose of rocuronium (0.6 mg/kg), produced intubating conditions comparable to those of a higher dose of 1.2 mg/kg, with no major hemodynamic variations or adverse effects. A study by Czarnecki et al. [16] involving 280 patients randomly allocated into two groups (one receiving 60 mg/kg of magnesium sulfate before RSI with fentanyl, propofol, and rocuronium (0.6 mg/kg) and the other receiving a placebo pre-treatment with normal saline), found that the magnesium-rocuronium combination provided comparable excellent intubating conditions to the saline-Succinylcholine group. Thus, there is substantial evidence supporting the effectiveness of magnesium pre-treatment in reducing onset time and providing excellent intubating conditions with rocuronium. Our study concluded that the mean duration of action of rocuronium was 40.04±3.61 minutes in group A and 25.92±3.9 minutes in group B. Magnesium does not prolong the duration of action of rocuronium to a clinically significant extent, ensuring favorable outcomes. In summary, this study demonstrated that pre-treatment with magnesium sulfate before rocuronium improves intubating conditions, allowing even lower doses (0.6 mg/kg) to produce excellent intubating conditions comparable to higher doses of rocuronium (1.2 mg/kg) or the standard intubating dose of succinylcholine, with no observed adverse effects. Magorian et al. conducted a study on 50 patients where they were randomly allocated into three groups, each receiving either rocuronium/vecuronium/succinylcholine during intubation and concluded that the onset time of 1.2 mg/kg of rocuronium was similar to that of succinylcholine but with a longer duration of action whereas a lesser dose of 0.6 mg/kg was similar in onset to vecuronium [17].

Queiroz Rangel Micuci et al. conducted a study to determine the role of magnesium sulfate on the duration of rocuronium-induced deep and intense neuromuscular blockade (NMB) and the period of no response to nerve stimulation. They reported that the median duration of deep NMB was 20.3 min and 18.3 min in the magnesium and saline groups, respectively. The median duration of intense NMB was 21.7 min and 0.0 min in the magnesium and saline groups, respectively. The median duration of the period of no response was 40.8 min and 28.0 min in the magnesium and saline groups, respectively. They concluded that magnesium sulfate increased both the duration of intense NMB and the period of no response. The duration of deep NMB was similar in the magnesium sulfate group and saline group [18].

Nirmal et al. conducted a prospective randomized double-blind controlled study to investigate the effect of magnesium sulfate pre-treatment on rocuronium priming in achieving rapid onset of intubating conditions. They reported that 120 patients were randomized into group R (n=60) and group MR (n=60). The magnesium and rocuronium priming group (group MR) had a significantly shorter onset time when compared to the onset time in the rocuronium priming group (group R). This resulted in significantly earlier completion of intubation in group MR than in group R. They concluded that the combination of magnesium sulfate pre-treatment and rocuronium priming accelerated the onset of neuromuscular blockade, improved rapid-sequence intubating conditions, and resulted in earlier endotracheal intubation when compared with rocuronium priming used alone [19,20].

Our study helps us conclude that magnesium pre-treatment reduced the onset time of rocuronium just like its priming dose, making it an excellent drug for use in rapid sequence intubation. It is clear from our study that rocuronium has all the potential to become a pioneer drug in RSI, making it a boon for use in trauma and burn patients where avoidance of succinylcholine becomes essential. This study thus helps us to prove the potential benefits of the new age drug rocuronium with a combination of magnesium potentiating its clinical effects similar to that of succinylcholine minus its side effects without any major adverse effects or hemodynamic instability, motivating us to use it more often in clinical practice. However, the high cost of rocuronium makes its accessibility difficult for the Indian population. Similarly, due to the unavailability of sugammadex in India, the longer effect of rocuronium in cannot ventilate cannot intubate situations can prove disastrous.

Limitations of our study include the absence of a control group and a small sample size. A large sample study with a control group is required to further validate our results. We have compared the results of our study with those of other studies that did not use magnesium and used rocuronium alone. It would be a more rational comparison if we had used a control group without magnesium in our study. Also, intraoperative awareness and postoperative sore throat can be monitored.

## Conclusions

Magnesium pre-treatment enhances the neuromuscular blocking effect of rocuronium, reducing its onset time and prolonging its duration of action. With the use of magnesium pre-treatment, the intubating dose of rocuronium can be decreased, which will avoid the use of higher doses of rocuronium.
